# Phylogenetic relationships of the HA and NA genes between vaccine and seasonal influenza A(H3N2) strains in Korea

**DOI:** 10.1371/journal.pone.0172059

**Published:** 2017-03-03

**Authors:** Jin Il Kim, Ilseob Lee, Sehee Park, Joon-Yong Bae, Kirim Yoo, Hee Jin Cheong, Ji Yun Noh, Kyung Wook Hong, Philippe Lemey, Bram Vrancken, Juwon Kim, Misun Nam, Soo-Hyeon Yun, Woo In Cho, Joon Young Song, Woo Joo Kim, Mee Sook Park, Jin-Won Song, Sun-Ho Kee, Ki-Joon Song, Man-Seong Park

**Affiliations:** 1 Department of Microbiology, the Institute of Viral Diseases, College of Medicine, Korea University, Seoul, Republic of Korea; 2 Division of Infectious Diseases, Department of Internal Medicine, College of Medicine, Korea University, Seoul, Republic of Korea; 3 Department of Internal Medicine, Hallym University College of Medicine, Chuncheon, Republic of Korea; 4 Department of Microbiology and Immunology, Rega Institute, KU Leuven–University of Leuven, Leuven, Belgium; Icahn School of Medicine at Mount Sinai, UNITED STATES

## Abstract

Seasonal influenza is caused by two influenza A subtype (H1N1 and H3N2) and two influenza B lineage (Victoria and Yamagata) viruses. Of these antigenically distinct viruses, the H3N2 virus was consistently detected in substantial proportions in Korea during the 2010/11-2013/14 seasons when compared to the other viruses and appeared responsible for the influenza-like illness rate peak during the first half of the 2011/12 season. To further scrutinize possible causes for this, we investigated the evolutionary and serological relationships between the vaccine and Korean H3N2 strains during the 2011/12 season for the main antigenic determinants of influenza viruses, the hemagglutinin (HA) and neuraminidase (NA) genes. In the 2011/12 season, when the number of H3N2 cases peaked, the majority of the Korean strains did not belong to the HA clade of A/Perth/16/2009 vaccine, and no Korean strains were of this lineage in the NA segment. In a serological assay, post-vaccinated human sera exhibited much reduced hemagglutination inhibition antibody titers against the non-vaccine clade Korean H3N2 strains. Moreover, Korean strains harbored several amino acid differences in the HA antigenic sites and in the NA with respect to vaccine lineages during this season. Of these, the HA antigenic site C residues 45 and 261 and the NA residue 81 appeared to be the signatures of positive selection. In subsequent seasons, when H3N2 cases were lower, the HA and NA genes of vaccine and Korean strains were more phylogenetically related to each other. Combined, our results provide indirect support for using phylogenetic clustering patterns of the HA and possibly also the NA genes in the selection of vaccine viruses and the assessment of vaccine effectiveness.

## Introduction

Influenza virus is a RNA virus that belongs to the family *Orthomyxoviridae* [1]. Four antigenically different viruses, the H1N1 and H3N2 subtypes of influenza A virus (IAV) and the Victoria and Yamagata lineages of influenza B virus (IBV), cause seasonal epidemics among humans [2, 3]. Vaccines can reduce seasonal influenza-related mortality and morbidity [4, 5]. Due to the continuous accumulation of genetic changes in the virus proteins most readily recognized by the immune system, a phenomenon referred to as antigenic drift [6], there is a need to frequently update vaccines in order to guarantee their effectiveness [7, 8]. Which viruses are likely well-suited for use as vaccine strains is announced by the World Health Organization (WHO) in February and September each year for the countries in the Northern and Southern Hemispheres, respectively [9]. These recommendations follow on a comprehensive process of surveillance and serological evaluation of thousands of contemporary virus strains in cooperation with National Influenza Centers of WHO member states and Collaborating Centers [10]. Yet, despite the assiduous efforts of WHO, there is sometimes a mismatch between the vaccine and circulating strains leading to reduced vaccine effectiveness [11], as was observed for the H3N2 virus during the 2011/12 H3N2 seasonal influenza [12–16]. Moreover, the number of viral isolates of seasonal influenza viruses and clinical manifestations defined in influenza-like illness (ILI) patients [17, 18] indicate that the H3N2 virus had more pronounced impact on influenza epidemiology in Korea during this season.

The degree of antigenic drift can be quantified with a hemagglutinin inhibition (HI) assay [19, 20]. Because HI assay results are key in deciding which lineages should be included in the next season's vaccine, it is critical that the HI assay results accurately mirror the true antigenic distance between the compared strains. However, for reasons that are not completely understood but that are likely grounded in different influenza infection and/or immunization histories, the HI assay results based on standard ferret antisera do not always agree with those obtained when human antisera are used [21, 22].

Recent theoretical and computational modeling developments spurred hopes for bypassing the difficulties characteristic for the *in vitro* determination of a strain's suitability as a vaccine candidate with *in silico* analyses that only capitalize on the virus genetic data [23–25]. A key criterion common to all *in silico* methods is how the candidate vaccine clusters among other circulating strains in a phylogenetic tree. Perhaps not surprising, these studies focus only on the HA gene. However, the NA gene encodes another surface glycoprotein of influenza viruses, which is one of the two main antigens that induce antibody-mediated immune responses [26, 27]. In addition, given that the functional balance between the HA and NA proteins that initiates and terminates the reproductive cycle of influenza virus infection, respectively, is of importance in terms of viral fitness [1, 28, 29], the phylogenetic clustering patterns of the NA gene should be also considered for the selection of vaccine viruses.

In this study, we first investigated the correlation between the ILI incidence rates and the number of influenza isolates in Korea during the 2010/11-2013/14 seasons. To scrutinize the positive correlation between the peak of ILI rates and H3N2 circulation in the 2011/12 season, we investigated the evolutionary and serological relationships between the HA and NA genes of vaccine and contemporary Korean H3N2 strains in the 2011/12 season as well as in the subsequent two seasons. We also determined which amino acid changes in the trunk lineages of HA and NA phylogenetic trees were positively selected.

## Materials and methods

### Viral genetic sequences

For the analysis focusing on the HA and NA clustering patterns of H3N2 stains during the 2011/12 season, Korean sequences from the 2011/12 season were obtained from viral isolates (n = 21) of nasopharyngeal clinical samples that were collected in Korea University Medical Center (KUMC) Guro Hospital, or were downloaded from the Korea Influenza Sequence and Epitope Database (KISED; http://influenza.cdc.go.kr/home/w) (n = 154). These were complemented with reference sequences of the 2009/10-2011/12 seasons downloaded from the databases of National Center for Biotechnology Information (NCBI, https://www.ncbi.nlm.nih.gov/genomes/FLU/FLU.html) (n = 451) and of Global Initiative on Sharing All Influenza Data (GISAID, http://platform.gisaid.org/epi3/frontend#4b03b7) (n = 8). The HA and NA data sets were aligned using MAFFT [30, 31]. The nucleotide lengths of the aligned HA and NA data sets were 987 (nucleotide residues 49–1,035) and 1,407 (nucleotide residues 1–1,407), respectively (S1 Data).

For the analysis focusing on the 2010/11 and 2013/14 clustering patterns, an additional 25 KUMC HA and NA sequences (total n = 46: 2011/12 season, 21; 2012/13, 3; and 2013/14, 22) were included along with 21 sequence sets of the 2011/12 season. Reference sequences were again obtained from the NCBI (n = 1,810) and GISAID (n = 178) databases. To remove a possible bias caused by a number of Korean sequences, however, the KISED sequences were not included in this analysis. As a result, a total of 2,034 full-length HA and NA sequences (S1 Data) were then aligned using the same MATTF program (v7.130b) [30].

### ILI incidence rates and the number of isolates of seasonal influenza viruses in Korea

Information of the ILI incidence rates and the number of seasonal influenza viruses isolates detected in Korea was collected from the weekly updates of KCDC reporting the nation-wide activity of influenza viruses [18]. The collected data of the 2010/11-2013/14 seasons were then summarized by virus type or subtype on a weekly basis from September (approximately week 35) through June (approximately week 26).

## Phylogenetic study and evolutionary rates

Evolutionary relationships of the H3N2 HA and NA genetic sequences were inferred with the Markov Chain Monte Carlo (MCMC) sampling method implemented in BEAST (v1.8.2) [32]. The substitution process was modeled with a GTR+I+Γ model [33]. A relaxed molecular clock with rates drawn from a lognormal distribution was specified [34], and the skygrid was set as a flexible tree prior [35]. MCMC chains ran for 2 x 10^8^ iterations and were sampled every 2 x 10^5^ iterations. 10% of the chain length was removed as burn-in. Convergence and mixing properties of at least two independent chains per gene were assessed in Tracer (v1.6) (http://tree.bio.ed.ac.uk/software/tracer/). Maximum clade credibility trees were built using TreeAnnotator (v1.8.2) and visualized using FigTree (v1.4.2) (http://tree.bio.ed.ac.uk/software/figtree/). For synonymous and nonsynonymous substitution rates, independent MCMC runs were additionally analyzed with the same prior setting using tailor-made scripts.

### Positive selection profiles

Positive selection profiles acting upon the HA and NA genes were first assessed using the web server of Datamonkey (http://www.datamonkey.org) [36] and confirmed using the HyPhy package (v2.2) [37]. Site-specific, positive selection profiles were then determined based on the estimated ratios of nonsynonymous (dN) to synonymous substitutions (dS) at every codon by the single likelihood ancestor counting method using a REV nucleotide substitution model [38]. The cutoff value was ‘*p* < 0.05’.

### Hemagglutination inhibition (HI) assay

For use in a HI assay, we isolated 12 H3N2 strains from nasopharyngeal clinical specimens collected from outpatients who visited KUMC Guro Hospital in the 2011–12 season. A H3N2 vaccine virus X-187 (NIBSC code: 11/108) was provided by Green Cross Corporation (Yongin, Republic of Korea). A vaccine prototype A/Victoria/210/2009 virus was provided by Korea Centers for Disease Control and Prevention (KCDC, Osong, Republic of Korea). X-187/HA:T228S was generated by reverse genetics using the genetic plasmids of X-187 that were constructed into an ambisense pDZ plasmid [39]. The pDZ/HA:T228S plasmid was prepared by a site-directed mutagenesis (Stratagene, La Jolla, CA) according to the manufacturer’s instruction. All the viruses were purified by a plaque assay in Madin-Darby canine kidney (MDCK) cells and propagated in 9–10 days old embryonated chicken eggs. The MDCK cell was obtained from America Type Culture Collection (Manassas, VA) and maintained with EMEM (Lonza, Basel, Switzerland). Using the plaque-purified and egg-propagated viruses, we analyzed viral genetic sequences after reverse transcription PCR. HA units of each virus were determined by a hemagglutination assay using 0.5% (v/v) turkey RBC (tRBC). For a HI assay, we used 80 post-vaccinated patient sera, which were collected after 4–5 weeks post-vaccination in KUMC Guro Hospital in the 2011–12 season. To remove nonspecific inhibitors [40], the sera were treated with receptor-destroying enzyme (Denka Seiken, Tokyo, Japan). The RDE-treated sera were then two-fold diluted serially in 25 μl PBS and mixed with 4 HA units of a virus (in 25 μl). After one hour incubation at 37°C, 50 μl of 0.5% (v/v) tRBC was added to each virus-sera mixture and incubated 30–45 minutes at room temperature. HI antibody titers of the sera were determined as the highest dilution that showed complete inhibition of hemagglutination in duplicate experiments. This study was approved by the Korea University Guro Hospital Institutional Review Board (approval number: KUGH 13210–002).

## Results

### Correlation between influenza-like illness rates and the number of seasonal influenza isolates during the 2010/11-2013/14 seasons

**Fig 1:**
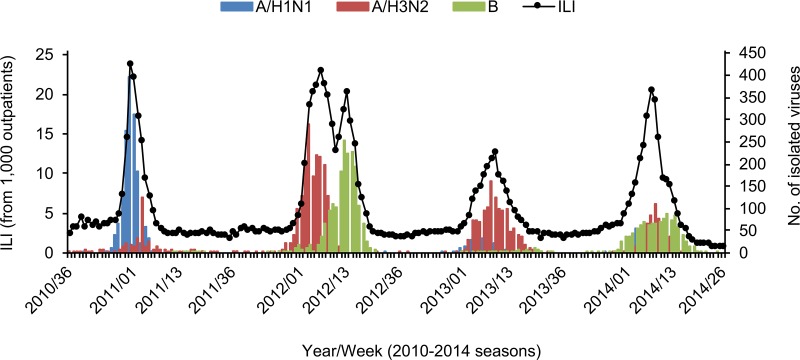
ILI cases and the number of isolates of seasonal influenza viruses in Korea during the 2010/11-2013/14 seasons. ILI cases per 1,000 patients and the number of isolates of seasonal influenza viruses were summarized based on the nation-wide weekly surveillance reports of KCDC on seasonal influenza activity in Korea during the 2010/11-2013/14 seasons. The weekly ILI incidence rates were indicated with black circles linked with a black solid line, and the weekly number of each viral isolates was indicated with different colors (for H1N1, light blue; for H3N2, light red; and for human B, light green).

Based on the weekly reports of influenza activity provided by KCDC [18], we correlated human ILI incidence rates with the number of typed seasonal influenza cases in Korea (Fig 1). This shows that the ILI incidence rates always peaked between January and March during the 2010/11-2013/14 seasons. Unlike the other three seasons, there were two ILI peaks in the 2011/12 season, which associate with the numbers of H3N2 and IBV isolates, respectively, in a chronological order. Of the three seasonal influenza viruses, the H3N2 virus was consistently detected in >20% of the typed isolates in the four consecutive seasons (S1 Table), whereas the fluctuation in proportion of H1N1 and IBV cases is more pronounced (Fig 1), and the persistent circulation of H3N2 appeared to impact the ILI incidence rates in Korea, especially during the 2011/12 season, as presented in the correlation coefficients (S2 Table).

### Phylogenetically discordant clustering patterns and serological comparison between the vaccine and Korean H3N2 strains from the 2011/12 season

**Fig 2:**
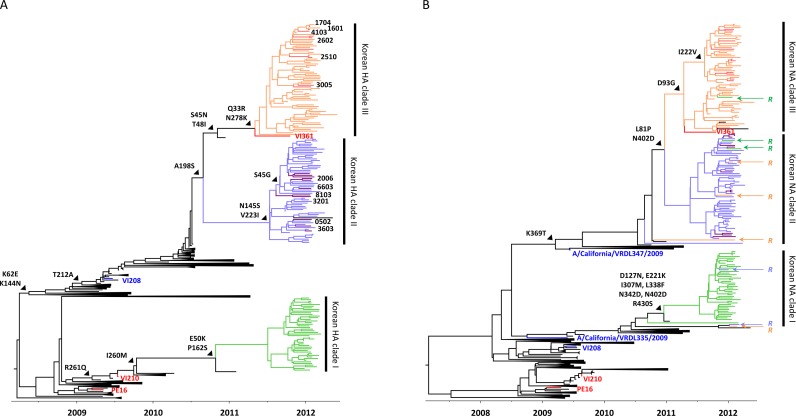
Phylogenetic relationships of the HA and NA genes of Korean H3N2 strains circulating in the 2011/12 season. The evolutionary relationships of the HA and NA genes of Korean H3N2 strains were reconstructed using the KISED (n = 154) and KUMC (n = 21) sequences. The sequences downloaded from the NCBI (n = 451) and GISAID (n = 8) databases were used as references. The three Korean HA and NA clades are indicated with different colors (clade I, light green; clade II, violet; and clade III, orange), and the KUMC sequences in the clades II and III were highlighted with purple and light red colors, respectively. The vaccine virus tips (PE16, VI210, and VI361) and the other clade-defining sequences (VI208, A/California/VRDL335/2009, and A/California/VRDL347/2009) were colored with red and blue, respectively. The KUMC viruses used for the HI assay were indicated with viral isolate numbers (please see Table 1). In the NA tree, the sequences of reassortment candidate strains between the HA and NA genes were indicated with a capital letter ‘*R*’ and the colors of their corresponding HA clades. Trunk lineage amino acid mutations were also indicated.

The evolutionary relationships between the vaccine and contemporary Korean H3N2 strains were investigated to better understand what might have caused the 2011/12 ILI peak. In the HA phylogenetic tree (Fig 2A), the KISED sequences of the 2011/12 season separated into three major Korean clades (Korean HA clade I, light green; Korean HA clade II, violet; and Korean HA clade III, orange), and the KUMC HAs contributed to the Korean HA clades II and III. Of the three Korean HA clades, only the clade I HAs belongs to the A/Perth/16/2009-like A/Victoria/210/2009 (VI210) vaccine virus lineage [41]. In contrast, the clade II and III HAs clustered into the subgroups of the A/Victoria/208/2009 (VI208) HA lineage (Fig 2A). Consistently with this observation, the selected KUMC strains, of which HAs were clustered into the Korean HA clades II and II, showed much lower antibody titers than three vaccine-related viruses in the HI assay using the post-vaccinated patient sera of the 2011–12 season (Table 1). Against the three vaccine-related X-187, X-187/HA:T228S, and VI210 viruses, the sera exhibited 239.88 ± 2.40, 211.12 ± 2.29, and 220.47 ± 2.29 HI titers, respectively. However, the same sera produced much lower titers (8.41 ± 1.78 to 33.35 ± 1.66) against the 12 selected KUMC strains, and more than 75 to 96% sera reacted poorly (less than 40 HI titers) (Table 1).

**Table 1:** HI-reactive antibody titers of post-vaccinated patient sera of KUMC Guro Hospital.

		Post-vaccinated sera of KUMC patients in the 2011–12 season		
Group	Virus	GMT (± SD)^a^	95% CI^b^	% ratio of ≥ 40 HI titer^c^
Vaccine	X-187	239.88 ± 2.40	199.53–292.12	100 (80/80)
	X-187/HA:T228S	211.12 ± 2.29	173.78–251.19	100 (80/80)
	VI210	220.47 ± 2.29	181.97–263.03	100 (80/80)
Korea HA clade II	KUMC1601	13.08 ± 1.74	11.48–14.79	6.25 (5/80)
	KUMC1704	9.02 ± 1.55	8.13–10.00	5.00 (4/80)
	KUMC2510	18.5 ± 1.58	16.60–20.42	17.50 (14/80)
	KUMC2602	15.69 ± 1.74	13.80–17.78	12.50 (10/80)
	KUMC3005	29.03 ± 1.70	25.70–32.36	48.75 (39/80)
	KUMC4103	18.18 ± 1.62	16.22–20.42	16.25 (13/80)
Korea HA clade III	KUMC0502	8.41 ± 1.78	7.41–9.55	3.75 (3/80)
	KUMC2006	16.25 ± 1.91	14.13–18.62	16.25 (13/80)
	KUMC3201	14.39 ± 2.00	12.30–16.60	13.75 (11/80)
	KUMC3603	33.35 ± 1.66	29.51–37.15	58.75 (47/80)
	KUMC6603	20.53 ± 1.74	18.20–23.44	25.00 (20/80)
	KUMC8103	10.72 ± 1.91	9.33–12.30	6.25 (5/80)

^a^Geometric mean titer (GMT) is indicated with standard deviation (SD).

^b^Confidence interval.

^c^The number of serum samples exhibiting more than 40 HI titer is indicated in parenthesis.

In the NA phylogenetic tree (Fig 2B), none of the Korean taxa clustered with the VI210 vaccine strain, either. Rather, the sampled Korean diversity appeared to be more closely related with the VI208 NA and grouped into three different clades (Korean NA clade I, light green; Korean NA clade II, violet; and Korean NA clade III, orange). Based on our sequence set, the clade I NA lineage appeared to evolve from the A/California/VRDL335/2009-like NA, and the clades II and III NA lineages from the A/California/VRDL347/2009-like NA (Fig 2B).

We could trace reassortment events for some Korean H3N2 strains. The HA and NA sequences from most isolates clustered in homologous clades, but three of the clade I HAs, two of the clade II HAs, and four of the clade III HAs clustered discordantly in the NA phylogeny (Fig 2B). None of these reassortant candidates retained mutations from aspartic acid to glycine or asparagine at NA residue 151 (D151G or D151N) (S1 Data), which was reported as a molecular signature for NA receptor binding variants of previous seasons [42].

### Different amino acid signatures between the vaccine and Korean H3N2 strains during the 2011/12 season

**Fig 3:**
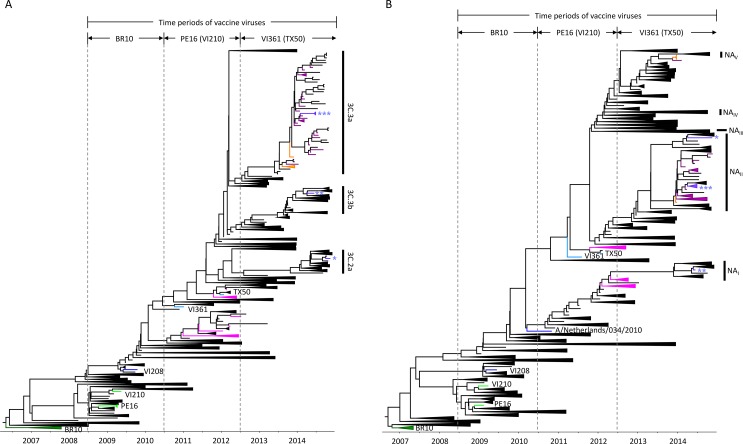
Clustering patterns of the HA and NA genes between the vaccine and circulating H3N2 strains during the 2008/09-2013/14 seasons. To compare the phylogenetic clustering patterns of the HA and NA genes between the vaccine and circulating H3N2 strains, the evolutionary relationships of the HA and NA genes were reconstructed using the genetic sequences of the 2008/09-2013/14 seasons. The vaccine virus tips (BR10, dark green; PE16 and VI210, light green; and VI361 and TX50, sky blue) and the 2011/12-2013/14 KUMC sequences are indicated with different colors: for 2011/12, pink; for 2012/13, orange; and for 2013/14, purple. The tips of clade-defining sequences were colored blue (VI210 and A/Netherlands/034/2010). For the three HA clades, each representative sequence was indicated with the different number of an asterisk (*, clade 3C.2a, A/Stockholm/29/2014; **, clade 3C.3b, A/Newcastle/22/2014; and ***, clade 3C.3a, A/South Australia/55/2014), and the same asterisk annotation was used for the NA of each corresponding strain. The five differentiated NA clades are indicated with NA_I_, NA_II_, NA_III_, NA_IV_, and NA_V_, respectively.

It had been previously reported that VI208 and the 2011/12 vaccine virus, A/Perth/16/2009 (PE16), are antigenically similar despite the considerable evolutionary divergence between their HA sequences [43]. There are, however, several indications that the 2011/12 Korean isolates do not *per se* share their antigenic properties with the VI208 and VI210 strains. Firstly, most of the amino acid mutations that influenced the differentiation of the three Korean HA clades from PE16, (Fig 2A) were found in HA antigenic sites, which determine the antigenic properties of Korean H3N2 strains (site A, K144N and N145S; site B, A198S; site C, S45G/N, T48I, E50K, and N278K; site D, T212A; site E, K62N, I260M, and R261Q; and others, Q33R and P162S) (Table 2) [44]. Second, the vaccine strain of the 2012/13 season (A/Victoria/361/2011 (VI361), which has a discrete antigenic profile from that of PE16 [45], clusters among the Korean H3N2 strains. This suggests that at least strains from this clade had the potential to evade the protective immunity raised in response to vaccination with the PE16-like VI210 vaccine. As the HA clade III isolates share their mutations in the antigenic sites with the clade II isolates (site A, K144N; site B, A198S; site D, T212A; and site E, K62E) (Table 2), the latter likely also exhibit antigenic similarity to VI361 rather than to the VI210 vaccine virus. In the 2012/14 seasons, KUMC strains appeared to harbor the HA gene of the 3C.3a clade (Fig 3A) and still retained 10 amino acid mutations, compared with the HA sequence of a WHO-recommended vaccine virus, A/Victoria/361/2011 (VI361). Of these, three mutations (Q33R, N145S, and N278K) had been already observed from the previous season, but the rest seven mutations appeared to be newly identified HA mutations (site A, A138S; site B, T128A, Q156H, F159S, and V186G; and site D, Y219S and N225D) in antigenic sites during the 2012/14 seasons (Table 2).

**Table 2:** Amino acid mutations associated with the Korean H3N2 clades.

		Amino acid mutation at a given residue	
Season	Clade	HA^a^	NA
2011–12	I	E50K (C), P162S, I260M (E), R261Q (E)	D127N, E221K, I307M, I338F, N342D, N402D, R430S
	II	S45G (C), K62E (E), K144N (A), N145S (A), A198S (B), T212A (D)	L81P, K369T, N402D
	III	Q33R, S45N (C), T48I (C), K62E (E), K144N (A), A198S (B), T212A (D), N278K (C)	L81P, D93G, I222V, K369T, N402D
2012–14	3C.3a	Q33R, T128A (B), A138S (A), N145S (A), Q156H (B), F159S (B), V186G (B), Y219S (D), N225D (D), N278K (C)	E221D, K258E, T329N, I392T

^a^HA antigenic sites (A, B, C, D, and E) are indicated in parenthesis.

Korean clade-related mutations were also found in the NA sequences in the 2011/12 season. Among the three Korean NA clades (Fig 2B), the highest number of amino acid mutations relative to the PE16 NA accumulated in clade I lineages (n = 7: D127N, E221K, I307M, I338F, N342D, N402D, and R430S), and the clades II and III harbored three (L81P, K369T, and N402D) and five (L81P, D93G, I222V, K369T, and N402D) mutations, respectively (Table 2). Of these, the I222V mutation identified in the clade III NAs has been associated with oseltamivir resistance of H3N2 virus [46]. During the 2012/14 seasons, only the four mutations (E221D, K258E, T329N, I392T) were found in the NA gene of KUMC strains, compared with the VI361 NA (Table 2).

We next looked for signatures of positive selection in the HA and NA gene sequences. This revealed that the HA antigenic site C residues 45 and 261, which associated with the divergence of Korean HA clades, were positively selected. NA residue 81, which was generated just before the separation of clade II and III lineages, appeared positively selected, also (Table 2). These may suggest that amino acid mutations in the HA antigenic sites and NA residues and evolutionary selection pressures working on them had affected the establishment of Korean H3N2 clades, which might eventually affect fitness differences between the vaccine and Korean H3N2 strains [25, 47].

### Phylogenetic relationships between the vaccine and circulating H3N2 strains during the 2008/09-2013/14 seasons

To investigate whether the `vaccine mismatch' clustering patterns of Korean strains were unique to the 2011/12 season, we also estimated the evolutionary relationships between the vaccine and other contemporary H3N2 strains over an extended time frame (2008/09-2013/14 seasons) (Fig 3). Three vaccine viruses had been recommended by the WHO during these periods: for the 2008/09-2009/10 seasons, A/Brisbane/10/2007 (BR10, dark green); for the 2010/11-2011/12 seasons, PE16 (and PE16-like VI210) (light green); and for the 2012/13-2013/14 seasons, VI361 (and A/Texas/50/2012 (TX50)) (sky blue) (Fig 3A and 3B) [43, 45, 48–51]. Even though the HA genes of vaccine viruses were always clustered with a previous clade, the picture emerges that, in the 2012/13 and 2013/14 seasons, which were characterized by lower levels of H3N2 cases when compared to the 2011/12 season (Fig 1), the vaccine and sampled Korean variants are more closely related to each other than in the 2011/12 season. (Fig 3A). There is no clear relationship between genetic and antigenic distance [20], but the vaccine update from VC361 to TX50 in the 2013/14 season appeared to decrease antigenic variations between the vaccine and Korean strains during these seasons. As this coincides with overall lower counts of H3N2 isolates, it might be said that the ILI peak in the 2011/12 season appeared to be mainly affected by the antigenic mismatch between the PE16-based vaccine and Korean H3N2 strains (Fig 1). It is also noteworthy that, contrary to what was seen in the 2011/12 season (Fig 2B), there were no clear discrepancies in the evolutionary distance between the vaccine and Korean strains in the NA segment history (Fig 3B). However, more divergent genetic clades were found in the NA than in the HA. Starting from the 2012/13 season, three main HA clades (3C.2a, 3C.3a, and 3C.3b) co-circulated until 2014 (Fig 3A). In the same time frame, there were five main NA clades (NA_I_, NA_II_, NA_III_, NA_IV_, and NA_V_) (Fig 3B). That all the 2012/13-2013/14 KUMC HA sequences belong to the 3C.3a HA clade, but that they associated with the NA_II_ and NA_V_ clades (Fig 3A and 3B) indicates a number of reassortment events previously occurred, similar to what was seen in the 2011/12 season (Fig 2B). All combined, our results indicate that phylogenetic discordant patterns have continuously been observed in the HA and NA genes between the vaccine and circulating H3N2 strains for the 2008/09-2013/14 seasons and that not only the HA genetic clade but also the NA should be accounted for in selecting influenza vaccine viruses.

## Discussion

Recent methodological advancements have placed phylogenetic inference in the spotlight as a promising tool for evaluating the antigenic evolution of influenza viruses from the genetic data. Unlike the classical HI assay, which uses post-infection animal and/or human antisera, phylogenetic analyses can capitalize on easier to generate, and hence more abundantly available data to achieve high prediction accuracy [52]. In this study, to explain the H3N2-driven increases of ILI incidence rates in Korea, we determined the evolutionary relationships of H3N2 HA and NA genetic sequences, the two main antigenic determinants of influenza viruses [26, 53]. Contrary to our thoughts, however, it was revealed that the HA and NA genes of the WHO-recommended vaccine viruses were all clustered into one of the various branches in previous seasons, not together with those of circulating strains in the corresponding vaccine seasons (Figs 2 and 3). These phylogenetic discordant clustering patterns between vaccine and circulating H3N2 strains (Figs 2 and 3) were supported by serological assay results. In the HI assay, the post-vaccinated patient sera exhibited less than 40 HI-reactive antibody titers against the selected KUMC strains, compared with the high HI titers against the vaccine-related viruses (Table 1). Based on these, vaccine effectiveness against H3N2 might be assumed so poor that the increases of ILI incidence rates appeared to be unavoidable during the 2011/12 season. Consistent with this observation, several reports of reduced vaccine effectiveness against the H3N2 virus have been reported from Asia, Europe, and North America [54–56] and even from Korea [15]. As demonstrated by the positive correlation between the ILI incidence rates and the number of H3N2 isolates (Fig 1 and S2 Table), the phylogenetic clustering patterns of the HA and NA genes might substantiate its significance with regard to the comprehensive process of vaccine virus selection.

In fact, a phylogenetically discordant clustering pattern had been already noticed between the HAs of PE16 and VI208 [43]. However, the HI assay results probably tipped the balance in favor of PE16 in the upcoming 2011/12 season [43]. For all that, the mutations in the three antigenic sites, such as K62E in site E, K144N in site A, and T212A in site D, which would be remarked between the HAs of PE16 and VI208, should have been considered for their potential effects on the change of viral antigenic properties (Table 2). In addition, of these three mutations, K144N was the one that could introduce N-linked glycosylation around the receptor binding site in the HA globular head region (S1 Fig). Given the effects of N-linked glycosylation on the antigenic property of influenza viruses [39, 57], the K144N mutation might transform viral antigenicity, and, a heavy glycan side chain anchored at residue 144 would probably interfere antibody binding targeting around this region, which would eventually skew overall HI assay results, in a biophysical perspective of HA binding to cellular receptor sialic acids. Moreover, the Korean H3N2 HA clade III viruses might harbor one more N-linked glycosylation in the antigenic site C residue 45 by a S45N mutation (Table 2).

Even though the mutation rates of the HA and NA genes in our study cannot be said higher than those of previous seasons (Table 3) [58], on-going genetic evolution that had been accumulated in circulating H3N2 strains since the WHO vaccine recommendation made in February 2011 might deepen the antigenic disparity between the vaccine and circulating H3N2 strains in the 2011/12 season. Currently, the recommendation of vaccine viruses is usually made at least six months prior to the corresponding influenza season to ensure that vaccine manufacturers can have enough time to prepare actual multivalent vaccines by securing the growth and immunogenicity of the vaccine viruses [59]. However, in the meantime, while the vaccines are being produced, many mutations can be accumulated, or a novel strain may newly appear. The replacement of previously circulating strains with this antigenically new strain may cause a situation such as the case of 2009 swine-origin H1N1 pandemic [60, 61]. In this case, the vaccines should be updated to meet the antigenicity of a novel strain, and this may cause a delay of vaccination schedules against seasonal influenza viruses. Otherwise, the vaccines are unlikely to provide optimal effectiveness in the end. Hence, to ensure that all the possible seasonal variants at a certain season are to be tested by both serological and phylogenetic clustering pattern evaluation, a current system of vaccine production using fertile chicken eggs should be reconsidered to reduce a vaccine production period and to remove a setback of egg-adaptive mutations [5, 62, 63].

**Table 3:** Evolutionary rates and selection profiles determined with the HA and NA genes of H3N2 strains circulating for the 2009/10-2011/12 seasons.

	Evolutionary rate (x 10^−3^ substitution/site/year)^a^			Selection profiles	
Gene	Nucleotide	Synonymous	Nonsynonymous	dN/dS	# of positive selection site
HA	4.84 ± 0.47	2.34 ± 0.02	2.15 ± 0.02	0.354	2 (45, 261)^b^
NA	3.87 ± 0.31	2.27 ± 0.01	1.42 ± 0.01	0.269	3 (44, 81, 151)

^a^Standard deviation of evolutionary rates is indicated with ‘± values’.

^b^Positive selection residues (H3N2 numbering) are indicated in parenthesis.

The NA is one of the two main antigenic determinants [26, 27, 53]. However, for the time being, the NA clade is not considered in the process of vaccine virus selection. If it had been considered, PE16 should have been never listed as a vaccine virus because all the Korean NA sequences in the 2011/12 season constituted subgroups under the VI208 NA, not under the PE16 NA (Fig 2B). Considering the persistently circulating HA and NA clades until 2014 (Fig 3B), it was again apparent why the NA clade should be taken into account for the selection of influenza vaccine viruses. Compared with the three HA clades (3C.2a, 3C.3a, and 3C.3b) (Fig 3A), the five NA clades (NA_I_, NA_II_, NA_III_, NA_IV_, and NA_V_) had evolved until 2014, and these might suggest the effects of various NA clades on the antigenic evolution of recent H3N2 strains, as indicated in the potential reassortment events between the HA and NA genes in some of 2011/12 Korean strains (Fig 2B). Indeed, the HAs of 3C.2a and 3C.3a clades appeared to be coupled with the NA_II_ clade NA (Fig 3B). Considered all together, our results suggest the importance of not only the HA phylogenetic clade but also the NA one with regard to the selection of vaccine viruses and the estimation of vaccine effectiveness along with the serological HI assay.

## Supporting information

S1 Fig Structural representation of HA amino acid mutations and the sequons of potential N-linked glycosylation at HA residues 45 and 144. (A) Based on the amino acid sequence of PE16 HA, amino acid mutations of the three HA clades (Fig 2A) were indicated with different colors (clade I, light green; common mutations in the clades II and III, magenta; clade II only, violet; and clade III only, orange) in a HA monomer of trimeric HA structure (PDB ID = 2HMG). HA regions were indicated as HA1 (light blue) and HA2 (light pink). The other two monomers were colored with dark grey. RBS, receptor binding site. (B) The two potential N-linked glycosylation sites that would be newly introduced at HA residues 45 and 144 were indicated with the amino acid signatures of their sequons, respectively (for N-linked glycosylation at residue 45, 45-46-47 and for N-linked glycosylation at residue 144, 144-145-146).
(TIF)

S1 Table The number of seasonal influenza virus isolates during the 2010/11-2013/14 seasons. (PDF)

S2 Table Pearson correlation coefficients between ILI cases and the number of seasonal influenza isolates in Korea. (PDF)

S1 Data Data sets of H3N2 HA and NA genetic sequences. (ZIP)
