# Supplementary figures and images for: Phylogenetic relationships of the HA and NA genes between vaccine and seasonal influenza A(H3N2) strains in Korea
Source: PLoS One. 2017 Mar 3;12(3):e0172059. doi: 10.1371/journal.pone.0172059 (PMC5336230; doi:10.1371/journal.pone.0172059)

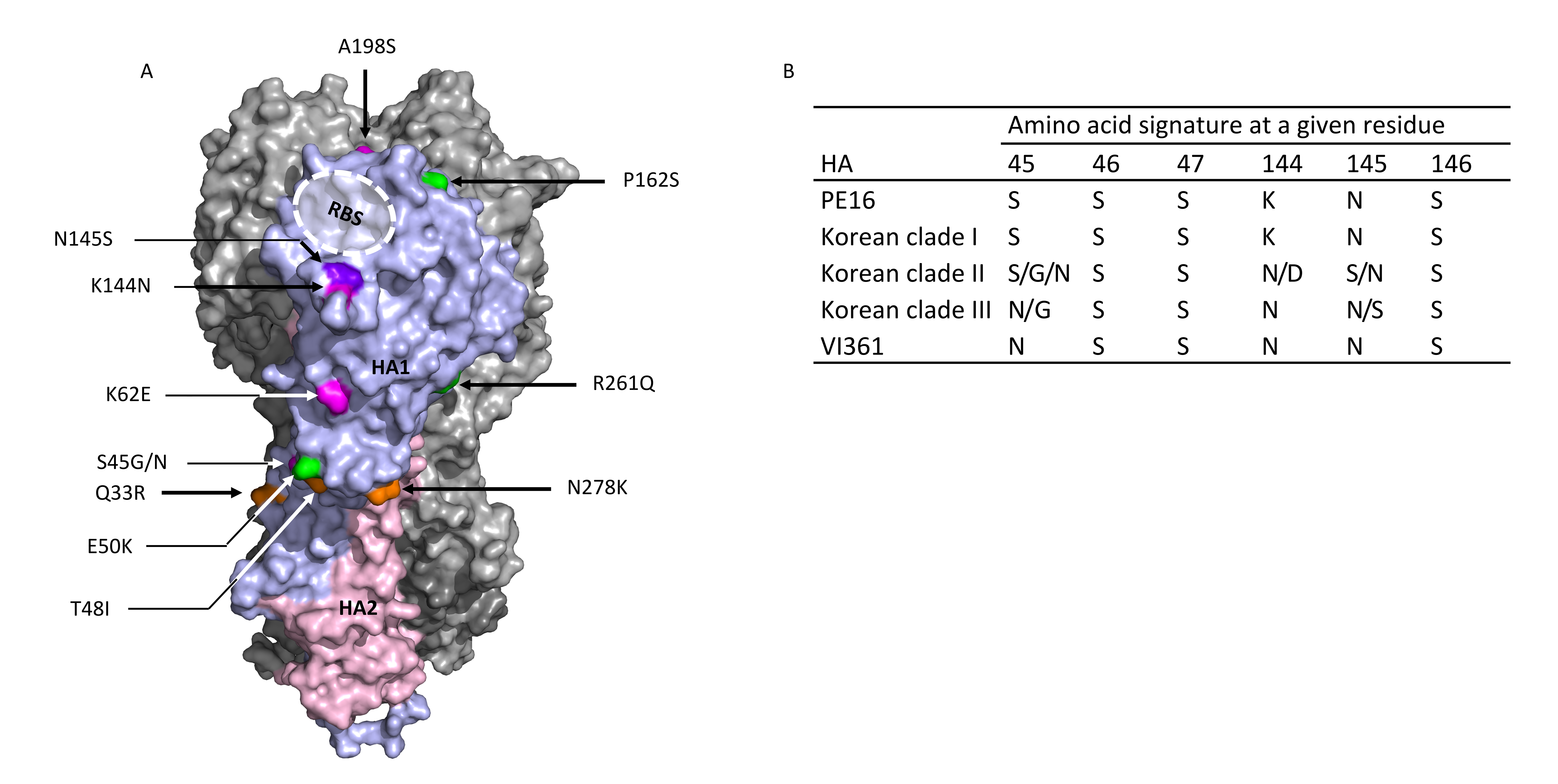

Supplement: S1 Fig — (A) Based on the amino acid sequence of PE16 HA, amino acid mutations of the three HA clades (Fig 2A) were indicated with different colors (clade I, light green; common mutations in the clades II and III, magenta; clade II only, violet; and clade III only, orange) in a HA monomer of trimeric HA structure (PDB ID = 2HMG). HA regions were indicated as HA1 (light blue) and HA2 (light pink). The other two monomers were colored with dark grey. RBS, receptor binding site. (B) The two potential N-linked glycosylation sites that would be newly introduced at HA residues 45 and 144 were indicated with the amino acid signatures of their sequons, respectively (for N-linked glycosylation at residue 45, 45-46-47 and for N-linked glycosylation at residue 144, 144-145-146). (TIF) [file pone.0172059.s001.tif]
