# Supplementary material for: Phylogenetic relationships of the HA and NA genes between vaccine and seasonal influenza A(H3N2) strains in Korea
Source: PLoS One. 2017 Mar 3;12(3):e0172059. doi: 10.1371/journal.pone.0172059 (PMC5336230; doi:10.1371/journal.pone.0172059)
Supplement: S1 Table — (PDF) [file pone.0172059.s002.pdf]

S1 Table. The number of seasonal influenza virus isolates during the 2010/11-2013/14 seasons.

	The number of viral isolates at a given season (%)			
Virus	2010/11	2011/12	2012/13	2013/14
H1N1	1,551 (78.69)	1 (0.03)	331 (19.52)	345 (16.62)
H3N2	405 (20.55)	1,935 (51.49)	1,270 (74.88)	628 (30.25)
B	15 (0.76)	1,822 (48.48)	95 (5.60)	1103 (53.13)
Total	1,971 (100)	3,758 (100)	1,696 (100)	2,076 (100)
