# Supplementary material for: Phylogenetic relationships of the HA and NA genes between vaccine and seasonal influenza A(H3N2) strains in Korea
Source: PLoS One. 2017 Mar 3;12(3):e0172059. doi: 10.1371/journal.pone.0172059 (PMC5336230; doi:10.1371/journal.pone.0172059)
Supplement: S2 Table — (PDF) [file pone.0172059.s003.pdf]

S2 Table. Pearson correlation coefficients between ILI cases and the number of seasonal influenza isolates in Korea.

	Correlation coefficient			
Virus	2010/11	2011/12	2012/13	2013/14
H1N1	0.921	n.d.[a]	0.726	0.693
H3N2	0.516	0.868	0.976	0.954
B	n.d.	0.988	n.d.	0.805

[a]Not determined due to the limited number of isolated cases of H1N1 (2011/12 season) or B (2010/11 and 2012/13 seasons) virus.
